# The Physical Activity and Redesigned Community Spaces (PARCS) Study: Protocol of a natural experiment to investigate the impact of citywide park redesign and renovation

**DOI:** 10.1186/s12889-016-3822-2

**Published:** 2016-11-14

**Authors:** Terry T. K. Huang, Katarzyna E. Wyka, Emily B. Ferris, Jennifer Gardner, Kelly R. Evenson, Devanshi Tripathi, Gabriel Martinez Soto, Matthew S. Cato, Jon Moon, Julia Wagner, Joan M. Dorn, Diane J. Catellier, Lorna E. Thorpe

**Affiliations:** 1 Center for Systems and Community Design, Graduate School of Public Health and Health Policy, City University of New York, New York, NY USA; 2 New York City Department of Parks and Recreation, New York, NY USA; 3 Department of Epidemiology, Gillings School of Global Public Health, University of North Carolina – Chapel Hill, Chapel Hill, NC USA; 4 MEI Research, Inc., Edina, MN USA; 5 City University of New York School of Medicine, Sophie Davis Biomedical Education Program, New York, NY USA; 6 Research Triangle Institute, Research Triangle Park, Durham, NC USA; 7 Department of Population Health, School of Medicine, New York University, New York, NY USA; 8 CUNY School of Public Health, 55 W. 125th Street, Room 803, New York, NY 10027 USA

**Keywords:** Parks, Recreation, Physical activity, Mental health, Natural experiment, Built environment, Planning

## Abstract

**Background:**

The built environment plays a critical role in promoting physical activity and health. The association between parks, as a key attribute of the built environment, and physical activity, however, remains inconclusive. This project leverages a natural experiment opportunity to assess the impact of the Community Parks Initiative (CPI), a citywide park redesign and renovation effort in New York City, on physical activity, park usage, psychosocial and mental health, and community wellbeing.

**Methods:**

The project will use a longitudinal design with matched controls. Thirty intervention park neighborhoods are socio-demographically matched to 20 control park neighborhoods. The study will investigate whether improvements in physical activity, park usage, psychosocial and mental health, and community wellbeing are observed from baseline to 3 years post-renovation among residents in intervention vs. control neighborhoods.

**Discussion:**

This study represents a rare opportunity to provide robust evidence to further our understanding of the complex relationship between parks and health. Findings will inform future investments in health-oriented urban design policies and offer evidence for addressing health disparities through built environment strategies.

## Background

Despite increased recognition of the role of the built environment in health, evidence for the relationship between parks (proximity or quality) and physical activity remains inconclusive, with research showing positive, negative or no effect [1–3]. Much of the research to date is observational, thus preventing conclusions of causality [3]. A few natural experiments examining the real-time effect of park renovations on physical activity and health demonstrate similarly inconsistent associations with park usage, physical activity, and other individual and community-level outcomes [4–8]. The lack of objectively measured physical activity and small sample sizes of many previous natural experiments also limit their validity, precision and generalizability [4–8].

The Community Parks Initiative (CPI) in New York City (NYC) provides a unique and unprecedented opportunity for the New York City Department of Parks and Recreation (NYC Parks) and the City University of New York Graduate School of Public Health and Health Policy (CUNY SPH) to conduct a large-scale natural experiment examining the prospective effect of park redesign and renovations on park usage, physical activity behaviors, psychosocial and mental health, and community wellbeing. CPI, a $285 million mayoral priority, is an equity-based park redesign and renovation project spearheaded by NYC Parks to improve under-resourced parks in underserved neighborhoods through: (1) redesigning physical structures and green spaces, (2) providing recreational programs, and (3) developing community partnerships [9]. NYC Parks has identified 134 parks with decades of under-investment and extreme capital needs in high-priority neighborhoods (≥20% poverty rate, ≥25% population growth in 2000–10, ≥110 people/acre). Forty-seven of the identified parks throughout NYC will be renovated in 2016–17.

The Physical Activity and Redesigned Community Spaces (PARCS) Study aims to evaluate the impact of park redesign and renovation on physical activity, park usage, perceived park quality, psychosocial and mental health, and community wellbeing. Using a longitudinal design (2016–2021), 30 intervention park neighborhoods will be compared to 20 intervention-eligible, socio-economically-matched control park neighborhoods with no plans for renovation during the study period. The protocol of the PARCS Study is described in this paper. We hypothesize that adult residents in intervention neighborhoods, relative to those in control neighborhoods, will demonstrate 1) a significant increase in total volume of physical activity, as measured by accelerometry, 2) significantly increased park usage and satisfaction, as measured by direct observation and self-reports, and 3) significant improvements in physical activity -related self-efficacy, stress, quality of life, social cohesion, social support for physical activity, and neighborhood satisfaction.

## Methods

### Study overview

**Fig. 1:**
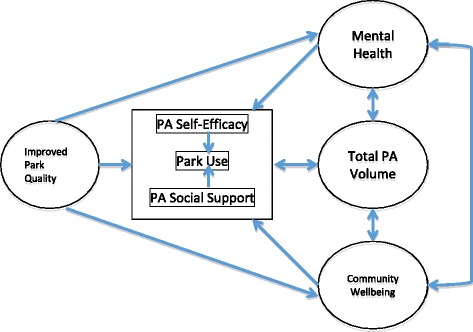
Study theoretical framework

**Fig. 2:**
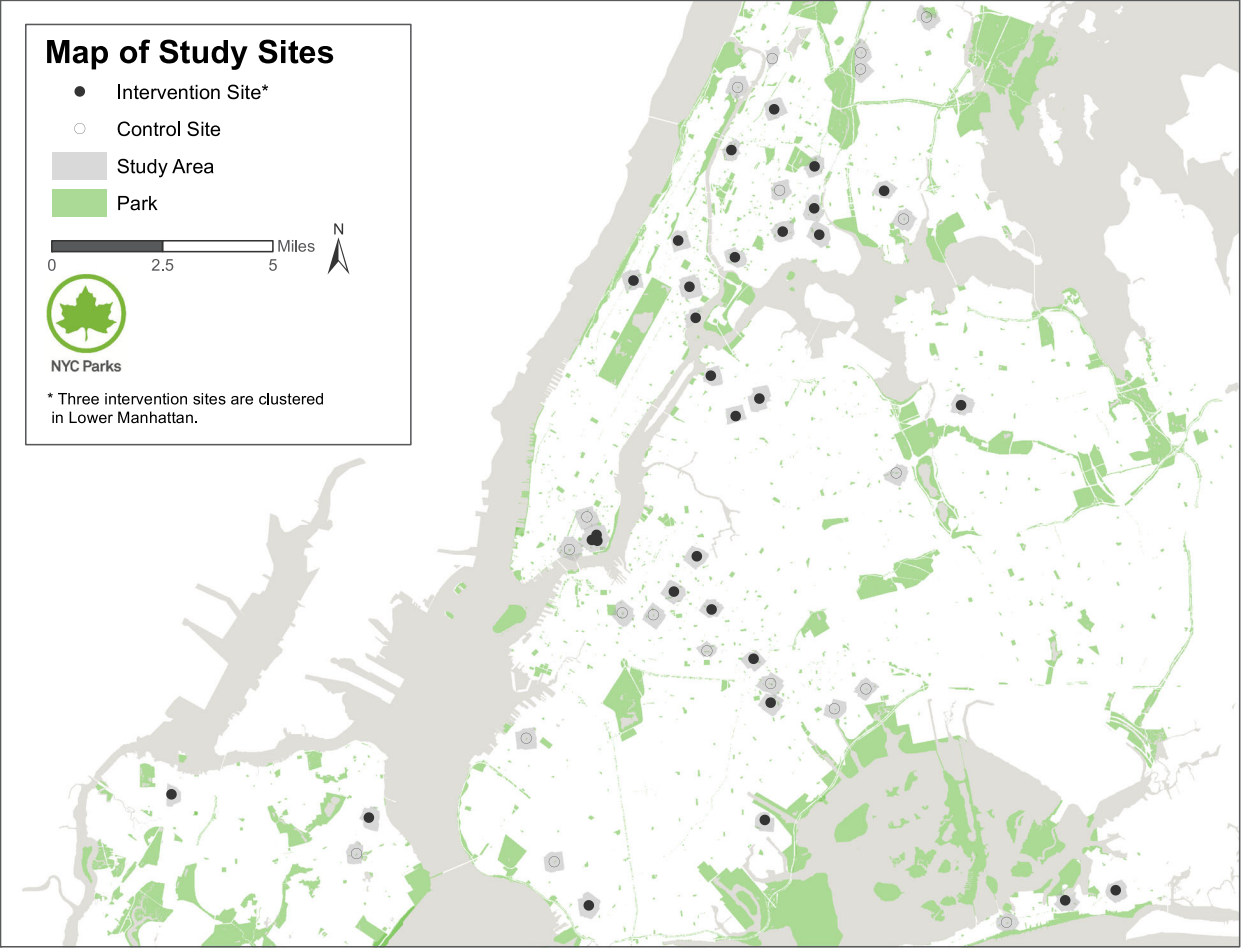
Map of study neighborhood sites

The PARCS Study is a prospective natural quasi-experiment with matched controls. The theoretical framework, adapted from environmental psychology, can be seen in Fig. 1. Our goal is to compare 3-year post-renovation outcomes between intervention and control neighborhoods. We aim to recruit and retain 780 participants in 30 intervention neighborhoods and 600 participants in 20 intervention-eligible, socio-economically matched control neighborhoods (total *n* = 1380, Fig. 2). Twenty intervention parks will close by Fall 2016 (Phase 1) and 10 additional intervention parks will close by Fall 2017 (Phase 2). Baseline data collection will occur from Summer 2016 – Summer 2017 (except in the middle of winter in January and February). There will be two follow-up assessments for accelerometry (1 year and 3 years post-renovation). Stress and quality of life will be measured annually at 3 time points post-renovation (1 year, 2 years and 3 years post-renovation). The remaining survey measures will be collected once at 2 years post-renovation only. Direct park observations will be conducted at baseline, and 1 year and 3 years post-renovation. See Table 1 for a schematic of study design and measurement timeline. The study is approved by the Institutional Review Board of the City University of New York.

**Table 1:** Study design and measurement timeline, Physical Activity and Redesigned Community Spaces (PARCS) Study – New York City

	2016		2017		2018		2019		2020		2021		Test
20 Intervention Parks (Phase 1) *N* = 520		A FS O				A SS O		FS		A SS O			Comparison of 3-year post-renovation outcomes
10 Intervention Parks (Phase 2) *N* = 260 (Total Intervention *N* = 780)				A FS O				A SS O		FS		A SS O	
					vs								
20 Control Parks *N* = 600		A FS O				A SS O		FS		A SS O			

Note. *A* accelerometry, *FS* full survey, *O* SOPARC (direct park observation), *SS* short survey (stress/quality of life). Shaded X = renovated parks reopening

Phase 1 and 2 intervention parks will be combined for analysis relative to control parks

### Study setting and population

CPI includes 134 high-priority and high-need parks for capital investment by NYC Parks. To be included, parks need to meet two of three selection criteria in each park neighborhood: high poverty (≥20% population below poverty line), high population growth (25% growth 2000–10), and high population density (≥110 people/acre). Thirty-five of these parks are part of Phase 1 of CPI (closure in late 2016) and 12 parks are part of Phase 2 (closure in late 2017); 30 of these parks have been selected for the intervention group (See Fig. 2). A sample list of the types of redesign and renovation is shown in Table 2. Another 20 CPI park neighborhoods with no renovation planned during the study period serve as the control group. Intervention and control park neighborhoods are selected based on best frequency matches (±6%) on key aggregated socio-demographic characteristics (Table 3). On average, intervention and control parks are 1.16 and 1.10 acres, respectively. For the purpose of this study, park neighborhoods are defined as the area encompassing 0.3-mile distance from the perimeter of each park given that most New Yorkers report walking up to 5 blocks to a park (unpublished data from NYC Parks). If a public housing complex straddles the border of such a buffer, the entire public housing complex is considered location-eligible.

**Table 2:** Comparison of existing (Baseline/Control) and renovated (Intervention) features

*Physical Elements*	
Existing	Renovated
• Out of date sports courts with cracked pavement and missing features	• Refurbished and reconstructed basketball, handball, tennis and other courts, including regulation sizes
• Asphalted play areas	• Synthetic turf conversions and multi-purpose fields
• Old-fashioned spray showers and mini pools	• Contemporary water feature elements
• Play equipment dating from 1960s–1990s	• Playground equipment meeting current safety and design standards, including ADA
• No or closed comfort station	• Refurbished or new construction comfort station meeting ADA standards
• Poorly configured benches and picnic tables in need of repair	• New tables, i.e. for chess, picnics; new benches and seating for passive recreation
• Minimal and unplanted horticultural beds and trees	• Increased plantings and horticulture
	• Adult fitness equipment
	• Performance and community event spaces
	• Skate park features
	• Green infrastructure components, i.e. rain gardens, bioswales, subsurface retention systems, permeable surfaces
	• Lower, more welcoming fences
	• New lighting fixtures
*Programming and Outreach Elements*	
Existing	Renovated
• Citywide and Borough-organized recreational programming	• Dedicated 6-month Playground Associates staffing all-day programs at capital renovation sites • Enhanced free adult fitness classes at sites • New partner programs, i.e. mobile library, running or bike classes, movie van
• Minimal or no direct community engagement specific to capital program sites	• Expanded outreach and community engagement staff dedicated to program neighborhoods
• Limited stakeholder engagement in capital process	• Held public scoping meetings to gather input on design program and park use with more than 1,100 attendees across 30+ sites

**Table 3:** Socio-demographic characteristics of study sites

	Citywide	Intervention Sites (*n* = 30)	Control Sites (*n* = 20)	Difference
Total population	8,175,133	860,098	515,626	344,472
Population over 18 years	78.4%	76.2%	76.9%	−0.7%
White residents	33.3%	18.5%	16.1%	2.3%
Black residents	22.8%	25.5%	31.6%	−6.1%
Asian residents	12.6%	14.2%	13.1%	1.1%
Hispanic residents	28.6%	39.7%	37.2%	2.5%
Population change	2.1%	3.6%	3.2%	0.4%
Population living below poverty line	20.3%	30.2%	27.6%	2.6%

Data in Table 3 are publicly available data

Given that CPI is focused on low-resource neighborhoods, the PARCS Study will target public housing residents to enroll in the study. NYC ranks first in the country for public housing accommodation [10]. A total of 599,493 (2016) New Yorkers are served by the New York City Housing Authority (NYCHA), representing 11.9% of the city’s rental apartments. NYCHA housing is spread throughout the city but concentrates in the lower-income neighborhoods featured in the current study. Families in NYCHA programs pay on average 30% of their family income for rent. Average family income is $23,672 and rent average is $483/month [10]. Because rent is highly subsidized, NYCHA residents represent a very stable population, an important factor for recruitment and retention.

### Inclusion and exclusion

Participants must live within designated NYCHA buildings or otherwise live within the 0.3-mile buffer of each study neighborhood. To maximize recruitment and minimize refusal, non-NYCHA residents can qualify for the study if they indicate they have lived in the neighborhood for at least two years, intend to stay in the neighborhood over the next four years or are otherwise engaged in a community organization (i.e., factors that improve chance of retention). We will include adults ≥18 years of age with no mobility problems and who understand/speak English, Spanish or Chinese (Mandarin or Cantonese).

### Participant incentives and retention

Participants will be offered $50 for each wave of data collection (up to 4 assessments total: baseline and 1, 2 and 3 years post-renovation). In addition, participants can receive an additional $10 per each successful referral (friend or neighbor but not family member from the same household). Participant retention is enhanced by the use of multiple strategies: 1) The sampling design around NYCHA or civically engaged residents increases the potential to retain and track participants over time in an urban environment known for its high levels of mobility; 2) Recruitment and outreach efforts are built into existing infrastructure and activities of *Partnership for Parks,* a community outreach organization supported by NYC Parks and the City Parks Foundation, which ensures regular and frequent touch points with participants throughout the year; 3) Designated study ambassadors will liaise and engage communities year-round; 4) We will provide participants with feedback by sharing early findings from some of their own data (data visualization) and give opportunities to partake in additional short but fun surveys throughout the year, with the potential to earn prizes if they engage; and 5) We will have the option to increase our incentive for later waves of data collection, as needed.

### Study protocol

Study staff will approach residents at each eligible NYCHA location to invite them to participate, and screen them for their eligibility using a mobile phone-based survey. If a participant is deemed eligible, study staff will register the participant through an app that is downloaded onto the participant’s mobile phone. The app, PiLR EMA™, is used to obtain electronic written consent from participants and provide the platform for time/location-triggered (e.g., park usage) and annual surveys (i.e., psychosocial questionnaires). The app is also GPS-enabled to track the participant usage of study parks. The participant will be given instructions to wear an Actigraph accelerometer (GT3X-BT, Pensacola, FL) for the next seven days. A second app, CentrePoint, will also be downloaded onto the participant’s phone, which enables the participants to upload accelerometer data at the end of each day for study staff to monitor wear compliance. A prepaid envelope will be provided to each participant to mail-return the accelerometers after 7 days of wear; unreturned devices after 2 weeks will be retrieved in person. An Android (v.4.3 or higher) or iOS (v.4 o higher) smartphone is required for data collection and study participation. According to the Pew Research Center, the digital divide among this audience is closing fast, and smart phone ownership, especially among Hispanics is at or over rates for other groups [11]. Daily phone/text reminders will be sent to participants to ensure accelerometer and survey compliance.

### Measures

Standard demographic information, smoking status [12], flu shot behavior [12], coffee consumption [13] (the latter two will be used as health risk behavior covariates and negative control outcomes [14]) will be collected in the screener or annual surveys in addition to the key outcome measures described below (see Table 4 for a summary of all measures).

**Table 4:** Key measures

*Construct*	*Variables* (*Sources*)
Physical activity (PA, total volume) *Primary outcome*	• Accelerometry data (ActiGraph GT3X-BT)
PA in study parks *Secondary outcome*	• GPS data (ActiPal^TM^ app)
Improvements in park features, programs and quality (direct observation) *Process/fidelity measures*	• SOPARC: accessible, usable, equipped, supervised, organized activity, dark, empty [18] • NYC Parks checklist: sustainability, accessibility, community health, safety and utility features, programming, partnerships • Park Quality Index: sustainability, beautification, community health, recreational options, accessibility, utility, safety
Park usage and engagement *Mediator*	• Direct observation through SOPARC: count of park users, gender, age, level of PA [18] • Self-reported reason for visit, visit length, mode of transport to park, visit companions, travel time, frequency of park usage in past 3 months, level of PA while at park in past 3 months [4] • Text-based self-reported diary of park usage
Park satisfaction and perception *Mediator*	• Accessibility, well kept, safety, ability to relax in park, ability to use for recreational purposes, walking distance, sufficient in neighborhood [24, 25] • Perception of neighborhood parks: overall quality, usage, attractive, safety, maintenance, shade, dog walking facilities, presence of gangs or vandalism, children’s interest in parks, time to walk to park, importance of particular park features for encouraging park-based physical activity [4]
Psychosocial/mental health *Secondary outcomes*	• Perceived Stress Scale [26] • Quality of Life Short-Form 12 [27] • Public Health Surveillance Well-being Scale [28] • Self-efficacy for Exercise Behaviors [29] • Social support for exercise behaviors [30]
Community wellbeing *Secondary outcomes*	• Social cohesion [33] • Perceived physical environment (Neighborhood Environment Walkability Scale) [35] • Contact with friends and neighbors [31] • Neighborhood Social Ties [32] • Sense of Community Index [34]
Demographic information *Covariates, moderators* *Negative controls*	• Age, sex, gender identification, income level, employment status, education level, marital status, number of children, size of household, language spoken at home, length of residency in neighborhood, smoking status, sexual orientation [12] • Flu shot behavior [12], coffee consumption [13] • flu shot behavior [57], coffee consumption [58]
Weather *Covariates*	• Daily high and low temperatures, humidity and rain/sun conditions

#### Physical activity

Physical activity will be measured using a 3-pronged approach over 7 consecutive days at baseline and at 1 and 3 years post park renovation: accelerometry for daily movement detection, GPS for location detection, and text-based self-reported physical activity behavior. The primary outcome is total volume of physical activity measured as average activity counts/min, consistent with the reported measure in existing literature on the built environment and physical activity. In addition, average vector magnitude (VM)/min and the same measures in 15-s epochs will also be examined (to leverage latest technology).

*Accelerometry:* Study participants’ physical activity will be measured by 7-day accelerometry using the ActiGraph GT3X-BT (Pensacola, FL), which provides activity counts for 3 axes (summarized as VM defined by adding the squared term for each of the three axes and then taking the square root of the sum) and steps taken. From these data we can estimate time spent engaging in sedentary behavior and in activity at different intensity levels (e.g., minutes of light, moderate, vigorous physical activity per day), and characterize individuals based on their pattern of physical activity. In addition, each of these indicators can be summarized as daily, weekday or weekend averages, by time of day (e.g., before/after traditional work hours), or during park use (as determined by accompanying location data). The placement and wear time protocol follows from prior experience in deploying accelerometers and analyses of accelerometry data [15]. The GT3X-BT has 4GB of data storage and a rechargeable battery capable of providing power for 25 days between charges. Accelerometry data will be downloaded and managed using CentrePoint and the ActiLife software from ActiGraph and integrated with spatial location data (see *Physical Activity Data Integration*). Accelerometers will be worn for 7 consecutive days annually on the right hip attached to an adjustable belt. Participants will be instructed to wear the monitors at all waking times except when bathing or swimming. Accelerometer non-wear will be defined by an interval of at least 90 consecutive minutes of zero VM counts/15-s, with allowance of up to 2 min of nonzero counts if no counts were detected during both the 30 min upstream and downstream from that interval [16]. Any nonzero counts (except the allowed short intervals) will be considered wear time. Counts in the non-wear period will be set to missing. To be included in the analysis, we will require ≥4 of 7 adherent days with an adherent day indicated by ≥10 h of wear.

*Smartphone-app for GPS:* Location detection will be measured using MEI Research’s smartphone-based PiLR EMA™ app (Edina, MN). PiLR EMA integrates the GPS function of Plot Projects™ and will track participants’ geo-spatial location throughout the same 7-day period as the accelerometer. Tracking is done via region-monitoring where park locations are preprogrammed into the app and identified when participants start and stop accessing the parks and their periphery (adjacent streets). This significantly reduces privacy concerns as we are not tracking residences, travel routes or other participant destinations outside of park locations. PiLR EMA geolocation can be assisted via cellular, WiFi and satellite connections, which is ideal for urban locations in which satellite connections may not always be available. Data will be time-stamped and synchronized with the accelerometer data, as described in *Physical Activity Data Integration*.

*Real-time text-based* physical activity *and park use survey:* Through time and location-triggered surveys on PiLR EMA, participants will record their daily physical activity behavior and park usage during the same time period as the accelerometer and GPS data are collected. Specifically, we will ask whether participants have used the neighborhood park and for what purpose (e.g., recreation or transit).

Physical activity *data integration:* Accelerometer and GPS data will be spatially and temporally integrated to understand the geo-spatial context of study participants’ physical activity patterns. The integration of the accelerometer and GPS data will allow us to identify and quantify the frequency, duration and level of study participants’ physical activity specifically during park visits or en route to/through parks. Readings from accelerometer and GPS devices will be normalized into a common measurement frequency to account for differences in device sampling frequency. Next, the readings will be aligned using interpolation and/or extrapolation to aggregate several short periods into a single long period or to separate a single long period into several small periods. Data then will be aggregated over 30-s epochs and classified into activity intensities [17]. We will investigate the applicability of different strategies to account for missing device readings such as using imputation or adjusting for time worn as part of analysis. Information collected through the GPS app also can be integrated with other GIS data sets for future research. We will integrate daily high and low temperatures, humidity, and rain/sun conditions based on zip codes of parks with individual physical activity data over the 7-day period.

#### Direct observations of park usage and habitual park usage

Park usage will be measured through both direct observation and study participant surveys. Using the System for Observing Play and Recreation in Communities (SOPARC), direct observations of aggregate park usage at baseline and 1 and 3 years post-renovation will measure the average number of park users at each study park as well as their gender, age and activity level. SOPARC is a validated direct observation tool that captures park characteristics and park users’ behavior [18]. In addition, study participants (not park users during SOPARC) will also provide self-reported measures of habitual park usage in annual surveys at baseline and at 2 years post-renovation via questions developed by Veitch et al. [4] These questions have demonstrated good test-retest reliability [4].

### Changes in park facilities, programs, engagement, and quality

Park improvements will be measured in three ways: 1) We will use SOPARC to document changes in park conditions and usership; 2) NYC Parks maintains a detailed checklist of park features, programming and partnership activities. Using this checklist, we will closely track changes in the physical structures and green spaces of parks as well as changes in programming and partnerships; and 3) To further measure the impact of CPI on park quality and to use park quality in analysis, we will develop a park quality index that serves as a composite quality score based on the following design features: sustainability, beautification, community health, recreational options, accessibility, utility and safety. The park quality index will be an observation tool at the park level representing the summary of all domain-specific indicators. We will adapt similar rating tools [19–21] to account for dimensions beyond previously used scales such as sustainability, programming and partnerships, all of which have been implicated as important attributes of park quality [22, 23].

### Park satisfaction and perception

Study participants’ satisfaction with and perception of parks will be measured at baseline and at 2 years post-renovation using previously validated items. To measure perceptions of neighborhood green space in general, we will use survey questions from the EURO-URHIS 2 project which measure the quality and access of green space in relation to psychological distress [24]. These survey questions were adapted from the validated Neighbourhood and Health Questionnaire used in the “Vitamin G” research study [25]. To assess participants’ perceptions of specific neighborhood parks, we will also use survey questions developed for a similar, though smaller-scale, park renovation natural experiment study [4]. The park perception questions developed by Veitch demonstrated good test-retest reliability [4].

### Psychosocial and mental health

To assess study participants’ psychosocial and mental health, the survey will include questions to measure stress, quality of life, physical activity self-efficacy, and social support for physical activity. The selected constructs have been widely used and validated in obesity and physical activity research. Mental health will be assessed through the Perceived Stress Scale [26], Short-Form 12 (SF-12) [27] will assess overall mental and physical health and the Public Health Surveillance Well-being Scale [28] will measure general wellbeing. Two additional scales, Self-efficacy for Exercise Behaviors [29] and Social Support for Exercise Behaviors [30], will be used. Stress and quality of life will be measured at baseline and each of the 3 follow-up visits. The other constructs will be measured at baseline and at 2 years post-renovation.

### Community wellbeing

Measures of community wellbeing draw on established constructs used to measure a range of community-level social indicators including social cohesion, perceived neighborhood environment, contact with neighbors and friends and neighborhood ties. We will use a measure of contact with neighbors and friends in the neighborhood initially used by Maas et al. in a similar study assessing the role of social contacts in the relationship between green space and health [31]. To measure neighborhood ties, we will use a survey developed by Kuo and colleagues to examine the relationship between neighborhood environments, including green spaces, and social ties in an urban public housing development [32]. In addition, we will use the social cohesion scale [33] and the sense of community index. [34] Finally, neighborhood satisfaction will be assessed using the eponymous subscale plus additional adapted items from the Neighborhood Environment Walkability Scale [35]. These measures will be taken at baseline and 2 years post-renovation.

### Analysis

This study uses a longitudinal and clustered quasi-experimental design with matched controls. General descriptive statistics (mean, median, standard deviation, inter-quartile range, minimum and maximum, proportion) will be calculated for all study variables at baseline and 1, 2 and 3-years post-renovation. Graphical displays (e.g., histograms and boxplots) will be produced and demographic characteristics of study participants will be examined. Park characteristics will be summarized for the intervention and control parks. To confirm that matching created approximately equal distributions of socioeconomic variables in the intervention and control parks, the two groups will be compared on these variables. The primary hypothesis of improved physical activity, park usage and psychosocial health in intervention vs. control neighborhoods will be analyzed using a difference-in-difference (DID) approach for repeated measures via mixed-effects models. Average activity counts/min indicating overall average physical activity will be the primary dependent variable (VM/epoch will be explored also). The DID model will include fixed effects for the intervention, post-renovation time-periods (1–3 years) and their interactions and will adjust for within-person and within-park correlations [36–38]. The intervention effect will be estimated by the interaction coefficients over time. The analysis will also be stratified by type of respondent when possible (e.g., male vs. female, above vs. below poverty line, etc.). All analyses will be adjusted for participant- and park-level covariates. Two-tailed alpha level will be set at *p* = .05. Sensitivity analyses will be conducted using alternative analytic approaches (e.g., models that make different assumptions about the homogeneity of variance over time). A separate analysis will be conducted with negative control outcomes (flu shot behavior/coffee consumption) to determine whether the intervention effects were specific to the outcome of interest. Analyses will be adjusted for multiple comparisons using the step-up method to control for the false discovery rate method [39].

We will explore different mediating and moderating pathways among park usage, physical activity -related self-efficacy and social support, physical activity behavior, mental health, and community wellbeing (Fig. 1). In addition, we will explore individual characteristics and health risk behaviors as potential moderators of the relationship between intervention and health outcomes. For these models, we will explore both fixed-effects models of change scores and mixed-effects models with repeated measures. Furthermore, to examine the change pattern of physical activity over time, we will use mixed-effects regression model with data from all intervention park neighborhoods. The model will include two random effects (intercept and slope) and a fixed effect for time. We will also test for various curvilinear trends over time to determine the trajectory of changes in physical activity behavior over time. We will compare models with AR1 terms with those of other error structures. Comparative evaluation of models will depend on the examination of Akaike’s Information Criteria and Schwartz’s Bayesian Criterion, which adjust the basic log likelihood results for complex models. We will examine patterns of missing data by pattern-mixture components in the mixed-effect models as needed [40].

### Sample size estimation

We propose to retain a total of 1380 individuals, which will be achieved by sampling 26 individuals in each of the 30 intervention (*n* = 780) and 30 individuals in each of the 20 control (*n* = 600) park neighborhoods. Anticipating an attrition rate of 25%, we will enroll a total of 1880 individuals (1080 intervention and 800 control), ensuring an adequate sample size for the primary analysis. Power analysis for this study was planned to provide adequate power (≥.80) to detect at least 50 counts/min difference from baseline to 3 years post-renovation between the intervention and control groups on change in the primary outcome (activity counts/min) with standard 2-tailed alpha = .05 [41]. This conservative difference of 50 activity counts/min corresponds to an annual increase of 219,420 total activity counts (TAC) based on an average of 53 min/park visit, 2.3 parks visits/week, and 9 usable park months/year [15]. Such an increase in TAC can be translated into significant improvements in several biomarkers, including HDL, triglycerides, plasma glucose, C-peptide, insulin, C-reaction protein, homocysteine and systolic blood pressure [42]. The change estimate of 50 activity counts/min stems from demonstrated built environment strategies such as light rail transit that mainly increases walking [41]; park interventions may lead to greater change because of both increased walking and exercise [15]. Based on prior studies of park users, we assumed the pooled subject-to-subject standard deviation of the primary outcome to be 200–300 activity counts/min (corresponding to effect size of. 17 -.25 standard deviation units) and an intraclass correlation within park of ≤ .02 (physical activity is less clustered than diet, smoking or demographic variables) [43]. For the secondary analyses, assuming a two-tailed alpha = .05, a sample size of 20 per neighborhood cluster with complete assessment data yield adequate power to detect relatively small effect sizes (.20 SD range for continuous outcomes and 10–20% difference in proportions), based on the assumption of relatively low ICC (≤.02). The moderator/mediator analyses are exploratory and will focus on the direction and magnitude of effects rather than statistical hypothesis testing.

## Discussion

The vast majority of research on parks and health outcomes has been cross-sectional in design [3]. Longitudinal studies tend to have small samples or, if larger in sample size, have only self-reported measures of environment and/or physical activity. Intervention studies are rare and also tend to be limited in scale. A recent systematic review of built environment interventions recommended more rigorous natural experiments [44]. To our knowledge, the PARCS Study represents the largest natural experiment to be performed with a longitudinal study design, matched controls, and objective measures of parks and physical activity. Long-term follow-up will also allow for the potential to observe any delayed effects or non-linear trajectory of change. In addition, most research to date has focused on the availability of or distance to parks in relation to health outcomes as opposed to park quality. However, one recent study from the UK suggests that the quality of parks can be just as important for mental health [24]. The current proposal will test specifically the effect of park quality improvement on multiple health outcomes.

Many Americans do not regularly engage in physical activity [45–47]. Based on self-reported data from the National Health and Nutrition Examination Survey (NHANES) 2011, 51.6% of adults meet the aerobic physical activity Guidelines for Americans of ≥150 min of moderate to vigorous physical activity per week [48]. Accelerometer-measured data from NHANES, however, reveals much lower levels, with fewer than 10% of Americans meeting the recommendation [46]. Though New York City (NYC) physical activity levels are slightly higher, with 29% of New Yorkers meeting recommended guidelines, high levels of physical inactivity persist. [49] Despite the city’s walkability, a recent study found that the average accelerometer-measured sedentary time for New Yorkers was 8.2 h per day [50]. Nationally and in NYC, physical inactivity contributes to health inequity as some groups, including black and Hispanic adults, older adults, less educated adults, and adults living at or near the poverty line, are less likely to meet physical activity guidelines than other groups [49, 51]. With many well-documented positive associations between physical activity and health, increases in physical activity could translate into overall improved health. Specifically, physical activity is associated with decreased risk for chronic diseases such as cardiovascular diseases, diabetes and some cancers, as well as prevention of weight gain and maintenance of weight loss [52]. Physical activity, even at low intensity such as walking, is also associated with improved mental health [53]. In the U.S., physical inactivity contributes to 6.7% of the burden of cardiovascular disease, 8.3% of the burden of type 2 diabetes, and 11% of all-cause mortality [54]. Given the significant health impacts and high levels of inactivity, increasing physical activity nationally and in NYC remains a priority.

There is limited research to draw on to determine the expected impact of built environment intervention on physical activity. In a light-rail/complete street intervention study, new riders of the light rail showed an increase in 48 ± 159 (mean ± SD) activity counts/min. [41] The impact of parks may be greater since parks can lead to both increased exercise as well as transit-related walking. In a study of residents across 5 cities in the U.S., park use was associated with an increase of 383 activity counts/min. [15] On average, residents visited parks 2.3 times a week, spending 53 min during each visit. Therefore, the cumulative impact of the increase in total volume of physical activity can be significant. Total activity counts have been shown to correlate more significantly with cardiometabolic risk factors than moderate-to-vigorous physical activity minutes and thus may be a particularly useful outcome measure to evaluate the effect of built environment interventions [42].

A substantial body of research recognizes the role of the built environment in promoting or inhibiting recreational physical activity and active transport behaviors [3]. Parks, in particular, may play a unique role in promoting physical activity. Evidence regarding the association between parks and physical activity, however, remains inconclusive. In Kaczynski’s review, nine studies reported significant associations between proximity to parks and walking [1]. However, existing research relies heavily on cross-sectional studies [3]. The use of inconsistent measures for physical activity and park access also limits the ability of existing research to provide more conclusive evidence [2]. Further research using a longitudinal approach with objective measures of physical activity and park characteristics is necessary to clarify associations between parks and physical activity. Beyond park proximity and usage, recent research suggests that park features, programming and overall quality may also impact physical activity behaviors [22, 55].

Research on the association between exposure to urban parks or green spaces and mental wellbeing has also largely come from cross-sectional studies and has shown mixed findings. Several recent studies in the US, Europe and New Zealand have corroborated on the positive association between proximity to parks or green space and global mental health [56–59]. However, two other studies showed null findings [60, 61]. Similarly, while some research has shown a significant inverse association of exposure to neighborhood green space with multiple aspects of mental ill-health, such as depression, anxiety and stress [62], other research has found this association to be limited only to some but not other aspects of mental health [63]. Of note, the effect of parks on mental health may be more pronounced in low-income neighborhoods. Using data from 34 European countries, one study showed that the socioeconomic inequality in mental wellbeing was 40% narrower among respondents reporting good access to green space [64]. Sturm and Cohen also reported that a nearby urban park was associated with the same mental health benefits as decreasing local unemployment rates by 2% [59]. These findings are important given the focus of the current proposal on park renovations specific to disadvantaged neighborhoods. Mechanisms for how parks or green spaces might influence mental health include increased physical activity [4, 65, 66], improved overall sense of quality of life [67] or increased social interaction and neighborhood social ties [31, 68]. Most of these pathways remain hypothetical, but the PARCS Study offers the opportunity to examine them empirically.

Although one of the hypothesized pathways of parks’ effects on health relates to enhanced community wellbeing, empirical research is limited. Since many parks function as meeting spaces or promote social interaction (especially in urban settings such as NYC), parks may have beneficial effects on community wellbeing such as social cohesion, social support or sense of community [31]. Initial research has provided mixed evidence for this relationship. One study found that while proximity to parks was associated with feeling less lonely, it was not associated with increased social contact or social support. [31] Kweon and Sullivan’s (1998) more focused study on older adults in urban environments reported a modest relationship between exposure to green outdoor space and neighborhood social ties and sense of community [69]. Of note, both studies indicated the strongest associations between parks and community wellbeing were among populations with low income or low education levels [31, 69]. Other research has demonstrated positive associations between park proximity and neighborhood social cohesion and human capital [70]. The success of one study in effectively building neighborhood capacity through active community engagement in park redesign is of particular relevance to this study as resident input has been central in the park redesign plan [71]. Strong community participation may increase the potential for desired impact on community wellbeing.

In conclusion, the PARCS Study is a rare opportunity to evaluate a key built environment intervention to improve individual and community health. The scale of CPI provides ideal conditions for a rigorous natural experiment design. Partnerships at the local and policy levels also offer tremendous promise for this project to truly influence policy both in NYC and beyond. Findings from this research will contribute to science on the built environment, community health and health disparities.
